# Exploring the learning curve of a new robotic microsurgical system for microsurgery

**DOI:** 10.1016/j.jpra.2022.09.002

**Published:** 2022-09-10

**Authors:** Carlotta Barbon, Lisanne Grünherz, Semra Uyulmaz, Pietro Giovanoli, Nicole Lindenblatt

**Affiliations:** Department of Plastic Surgery and Hand Surgery, University Hospital Zurich, Zurich, Switzerland

**Keywords:** Microsurgery, Robotic surgery, Robotic microsurgery, Symani, Lymphatic surgery, Robotic-assisted surgery

## Abstract

Robotic systems have recently been introduced into micro- and supermicrosurgery showing potential benefits for reconstructive surgery. After showing the feasibility and safety of using the Symani Surgical System® for lymphatic microsurgical procedures in humans, we present the results of the first twenty-two patients operated with the robot. The main goal of the study was to determine the learning curve using the Symani Surgical System® . In addition, we aimed at exploring the potential of robot-assisted anastomosis for lymphatic, free flap, and nerve reconstruction and defining the advantages and drawbacks of implementing the system into our daily routine. The operating times were compared between robotic-assisted and hand-sewn anastomoses. Moreover, outcomes and complications were recorded. In this first patient series, anastomotic times were significantly faster with the hand-sewn technique (14.1±4.3 min) when compared with the robot-assisted technique (25.3±12.3 min; p<0.01). However, the learning curve was very steep, and the time needed to perform the anastomosis has been consistently decreasing over time to the point where in the last operations, the times to perform particularly lympho-venous anastomoses were comparable between the two groups. Based on our experience up to date, robot-assisted surgery shows a promising potential in opening up new frontiers in reconstructive microsurgery, e.g., the reliable performance of anastomoses on even smaller blood and lymphatic vessels or on structures deeper within the body cavities, e.g., the thoracic duct.

## Introduction

Microsurgery represents a fundamental technique in reconstructive surgery, which is employed in many different fields, such as free flap reconstruction, nerve surgery, or lymphatic surgery. Anastomotic patency is largely dependent on the operator`s skills and experience. Over the last decade, new tools were developed to aid surgeons in performing complex procedures, among them robotic surgical systems. Recently, robot-assisted microsurgery was introduced into reconstructive surgery. 1, 2, 3, 4

In the past years, a lot of effort has been made to deliver clinically applicable systems. In 2020, the MUSA robot (MicroSure, Eindhoven, The Netherlands) was introduced. This device is equipped with fixed joysticks, which are connected to a scaffold and standard microsurgical instruments; however, this setup leaves the surgeon in a fixed position. ^1^ Subsequently to overcome this issue, the Symani Surgical System® (Medical Microinstruments, MMI, Calci, Italy) was introduced, which is the only system that offers wristed micro-instruments up to date. It consists of flexible robotic arms that can reach into deeper anatomical regions. The surgeon is able to perform movements that are translated into the operative field, while comfortably sitting on the platform chair. It provides several valuable features including motion scaling enhancing the surgeon's precision and dexterity. Visualization is achieved either with a conventional microscope or with a 3D visualization system at the surgeon's discretion. We have recently shown the feasibility and safety in performing micro- and supermicrosurgical anastomosis in lymphatic reconstruction with the system.^2^ The Symani Surgical System® was also used for a free flap in a post-traumatic upper limb reconstruction by Innocenti and his team^5^.

Therefore, it was the aim of this study to analyze the learning curve as well as the advantages and drawbacks of implementing the robot into micro- and supermicrosurgery on a daily basis. Furthermore, we aimed to explore the potential of microsurgical robot-assisted anastomosis for reconstructive surgery focusing on lymphatic surgery, free flap, and nerve reconstruction and record complications.

## Materials/Patients and Methods

**Figure 1:**
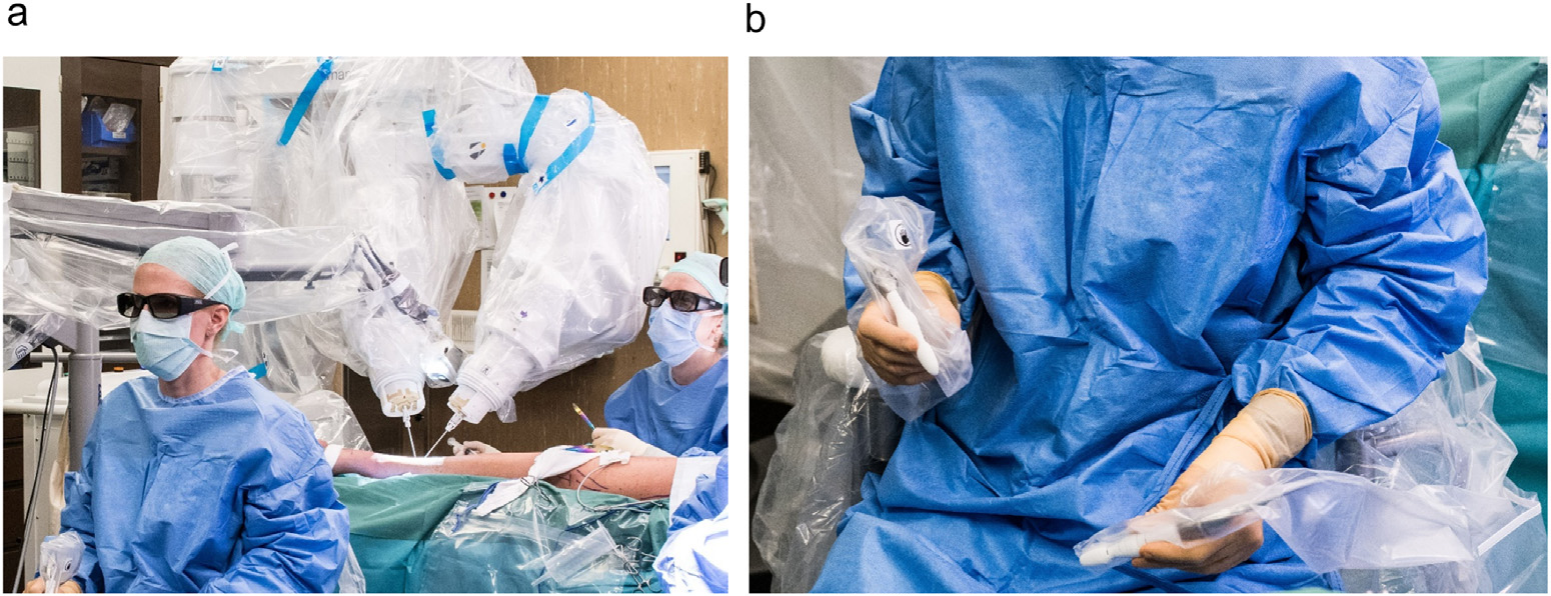
(a) Intraoperative set up of the Symani Surgical System® using a 3D exoscopic visualization system. (b) The surgeon controls the robotic system with two sterile manipulators and foot pedals. Hand movements are converted, and motion is scaled (7-20x) into movements of the robotic arms.

We conducted a retrospective mono-center study at the Department of Plastic Surgery and Hand Surgery of the University Hospital Zurich. The study was approved by the Cantonal Ethics Committee of Zurich (BASEC approval number 2021-02351). Between August 2021 and April 2022, 22 patients who underwent a microsurgical procedure with the Symani Surgical System® (Figure 1) and who gave written consent were included. Patients received either lymphatic reconstructive surgery, free flap reconstruction, or nerve coaptation. All surgeries were performed by the senior author. The operation records were analyzed regarding the following criteria: patient characteristics, type of surgery performed, time to perform the anastomosis, number of stitches, suture size, patency of the anastomosis, and complications. Particular attention was directed toward the evaluation of the learning curve. Data were analyzed using Microsoft® Excel Version 14.3.6. (Microsoft Corp., Redmond, WA, USA) and GraphPad Prism Version 7.04 (GraphPad, La Jolla, CA, USA). For the comparison of continuous parametric data, an unpaired t-test was performed. A p-value of < 0.05 was defined as significant.

## Results

Twenty-two patients underwent microsurgical procedures with the assistance of the Symani Surgical System® . Of those, eighteen patients received lymphatic surgery to address lymphedema, to treat lymphatic fistulas, or as a preventive measure after tumor removal. The remaining procedures were epineural coaptation, breast reconstruction with a profunda artery perforator flap (PAP), facial reanimation with a gracilis neurovascular flap, and lower extremity reconstruction with a superficial circumflex iliac artery perforator flap (SCIP) after removal of a vascular malformation. Most of the patients (n = 17) were operated on the lower extremity and inguinal region, while others received surgery at the upper extremity, torso, genitals, face, and neck. (Table 1)

**Table 1:** Patients and surgery characteristics

	n = 22	
**Age (years, mean ± SD)**	46.1	(± 19.2)
**Type of surgery n (%)**		
Lymphedema	13	(59%)
Nerve epineural coaptation	2	(9%)
LVA after tumor excision	4	(18%)
Flaps	2	(9%)
Lymphatic fistulas	1	(5%)
**Affected anatomic region n (%)**		
Upper extremity	1	(5%)
Lower extremity/inguinal region	17	(77%)
Genital	1	(5%)
Face/neck	2	(9%)
Trunk	1	(5%)

**Figure 2:**
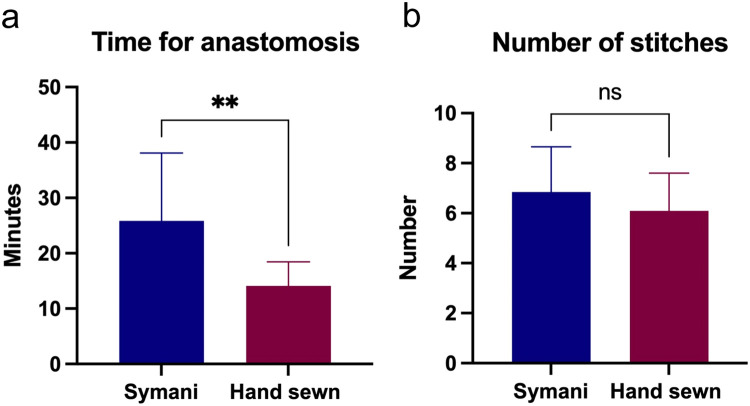
**(a)** Anastomotic times for robot-assisted anastomosis (Symani) vs. hand-sewn anastomoses. The mean time needed to perform the anastomosis with the robot was 25.3±12.3 min and for the hand-sewn procedures 14.1± 4.3 min (**p<0.01). (b) Number of stitches to perform the anastomoses were comparable between the groups (6.8± 1.8 vs. 6.1± 1.5; p=n.sig).

**Figure 3:**
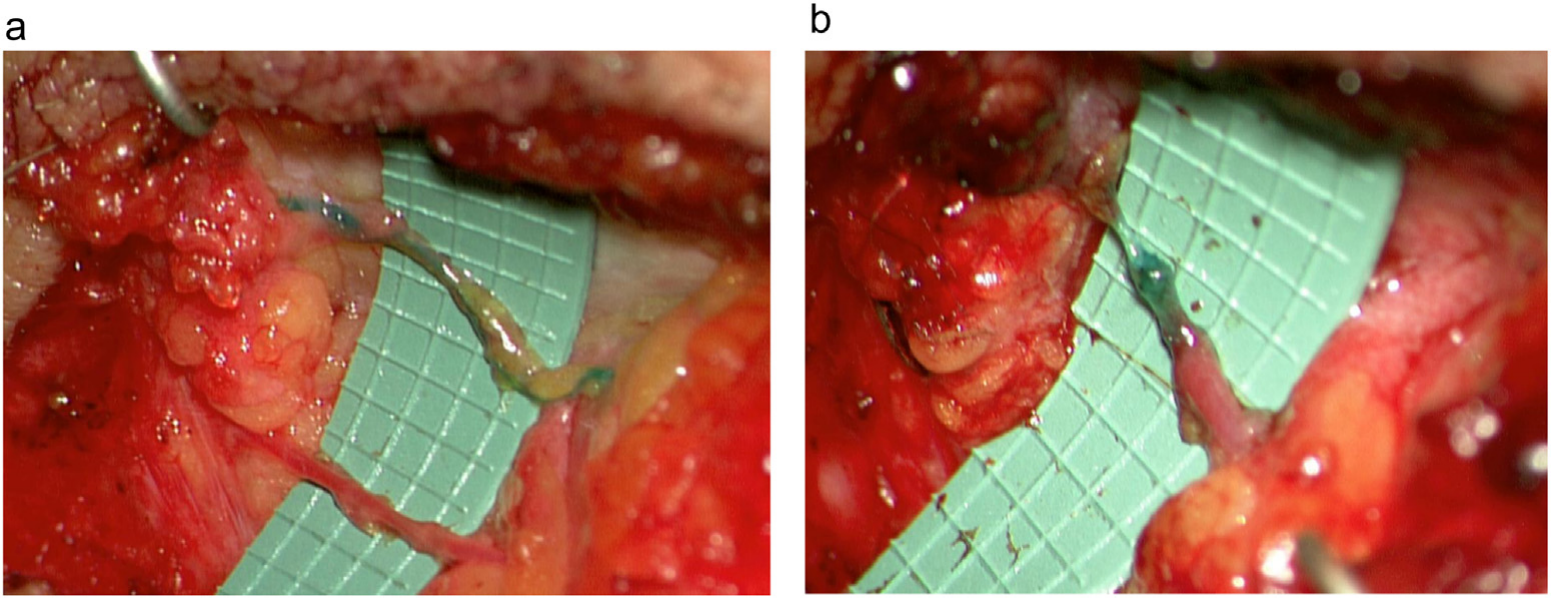
**(a)** For the treatment of genital lymphedema, a LVA between a very fine lymphatic vessel of 0.3 mm and a vein of 0.9 mm had to be performed by hand suture due to the fragility of the vessel. (b) Patent blue angiography shows a patent anastomosis.

Overall, 32 anastomoses were performed using the Symani Surgical System®. The most frequent were lympho-venous anastomoses (LVA) (n=20) and arterial anastomoses (n=9), either performed for free flap transfer or for free vascularized lymph node transfers (VLNT) (Table 2). Other procedures performed were lympho-lymphatic anastomoses (LLA) and epineural coaptations. For 84% of the cases operated with the assistance of the robot, the suture size used was 11-0 (Table 2). The mean time needed to perform the anastomosis with the robot was 25.3±12.3 min and for the hand-sewn procedures 14.1± 4.3 min (p<0.01). The mean number of stitches used for the robot-assisted procedures was 6.8± 1.8 and for the hand-sewn technique 6.1± 1.5 (p=n.sig) (Figure 2). A total of 97.5% of anastomoses were patent and either tested by ICG, patent blue, or clinically by observation and milking test. One thrombosis was recorded using the Symani Surgical System® on an irradiated vessel of the thorax during breast reconstruction. Procedural evaluation yielded that the thrombosis most likely occurred due to holding the vessel lumen with the dilator resulting in uncontrolled force application and intima damage. Therefore, direct manipulation of the vessels, in particularly the intima, should be performed with caution using the Symani Surgical System®, since there is no haptic feedback. Instead, vessels should only be lifted indirectly holding on to the adventitia or spread with the dilator to facilitate stitching. Save one all attempted anastomoses could be successfully performed with the robotic system. In one case of genital lymphedema, a 0.3 lymphatic had to be sutured by hand to a 0.9 mm vein due to better sensation when stitching the extremely thin vessel wall (Figure 3).

**Table 2:** Robot- and hand-assisted anastomosis and suture size

Variable	Robot-assisted anastomosis		Hand-sewn anastomosis	
	(n)	%	(n)	%
**Type of anastomosis**				
Lympho-lymphatic	1	(3%)	0	(0%)
Lympho-venous	20	(63%)	8	(73%)
Arterial	9	(28%)	3	(27%)
Epineural coaptation	2	(6%)	0	(0%)
**Suture size**				
9-0	4	(13%)	1	(9%)
10-0	4	(13%)	0	(0%)
11-0	24	(84%)	10	(91%)

**Figure 4:**
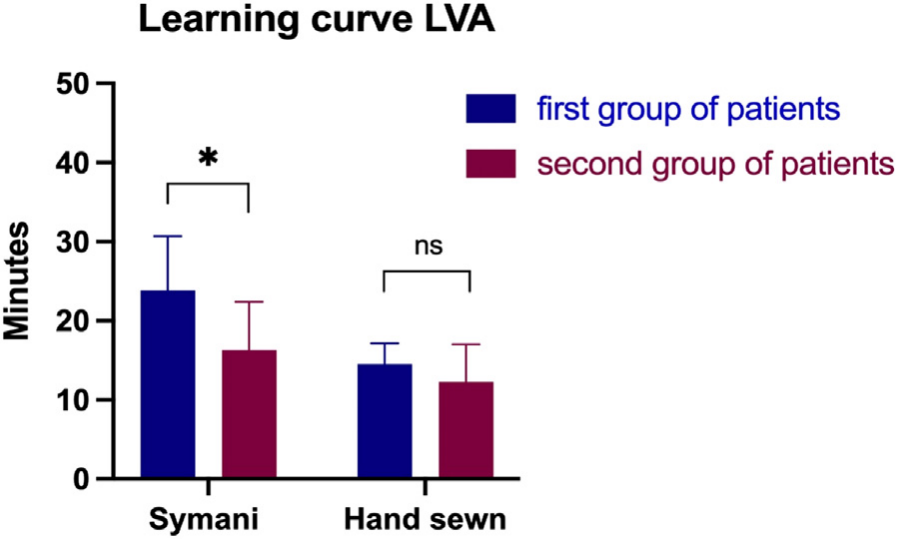
Comparison between anastomotic times in the first patient group (Aug to Dec 2021) and the second patient group (Jan to Apr 2022). Time to perform the LVAs significantly dropped from 23.9± 6.8 min in the first group to 16.3±6.1 min in the second group (*p<0.05).

**Figure 5:**
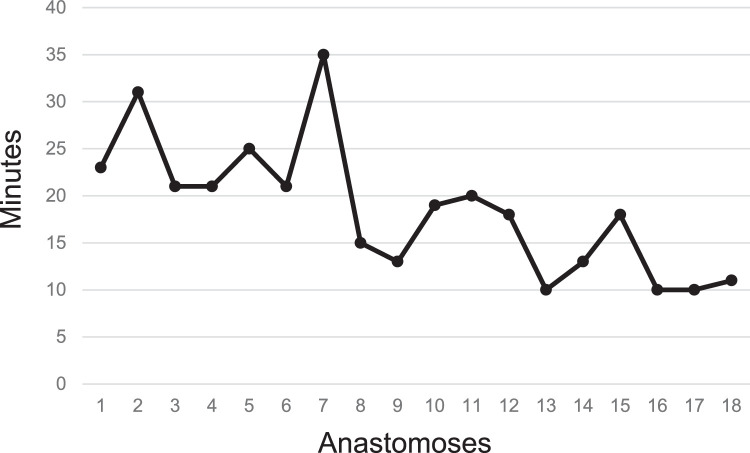
Anastomotic time for 18 consecutive robot-assisted LVAs show a significant decrease in the last cases reaching comparable times to hand-sewn anastomoses.

We observed a significant improvement when performing LVAs with the robotic system between the first and the second patient groups indicating a steep learning curve (Figure 4). Time to perform the LVAs significantly dropped from 23.9± 6.8 min in the first group to 16.3±6.1 min in the second group (p<0.05). The longest time to perform an anastomosis was 35 min with the hand technique vs. 59 min with Symani Surgical System®. Reasons for this long robot-assisted anastomotic time were technical issues with the system and a pronounced stickiness of the early instruments. During later procedures, stickiness was significantly reduced in new instrument badges and does not represent a major issue in current procedures. In the last surgical procedures, the time for LVA was comparable with the hand-sewn technique. For example, in the same patient, two LVAs were performed with the Symani and one with the hand-sewn technique; 12 and 10 min were required for the former and 10 min for the latter (Figure 5). The quickest anastomosis performed with the hand was in 8 min and with the robot in 10 min.

**Figure 6:**
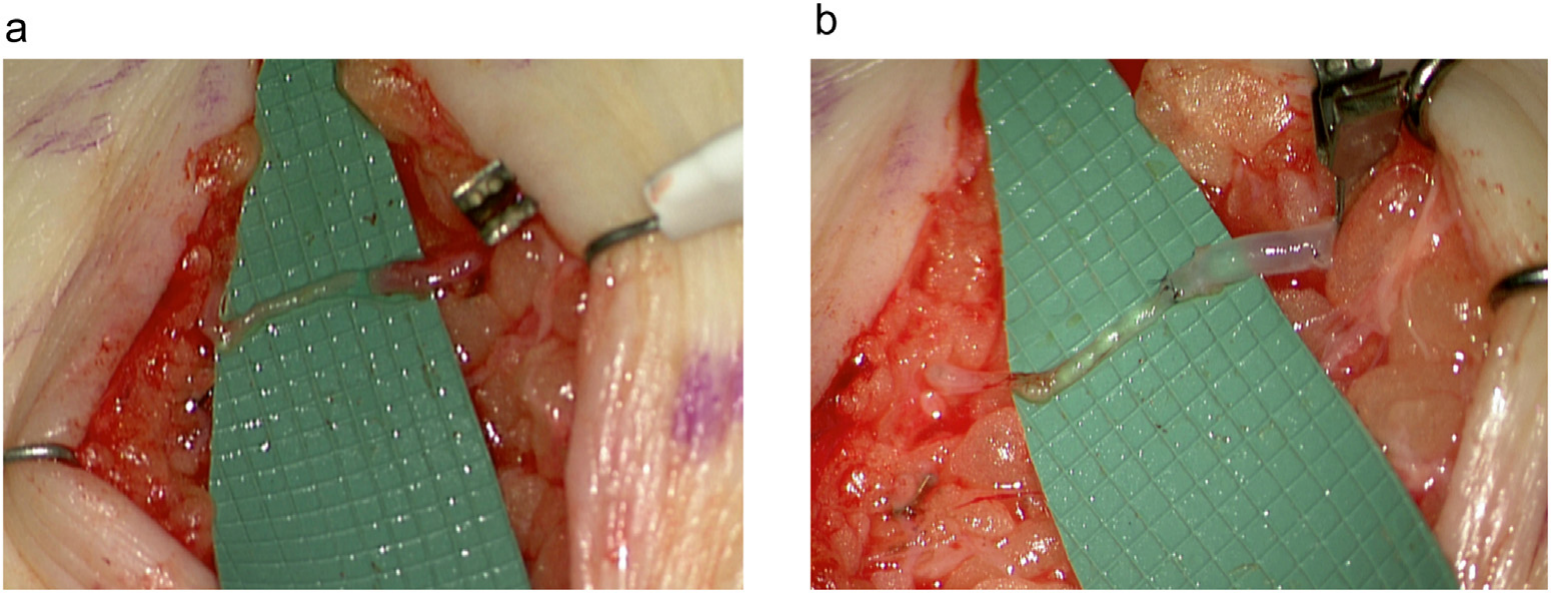
(a) Robotic-assisted LVA of a significant size mismatch between lymphatic vessel (0.7 mm) and vein (1.2 mm). (b) A marked lymphatic flow is detected crossing the anastomosis.

**Figure 7:**
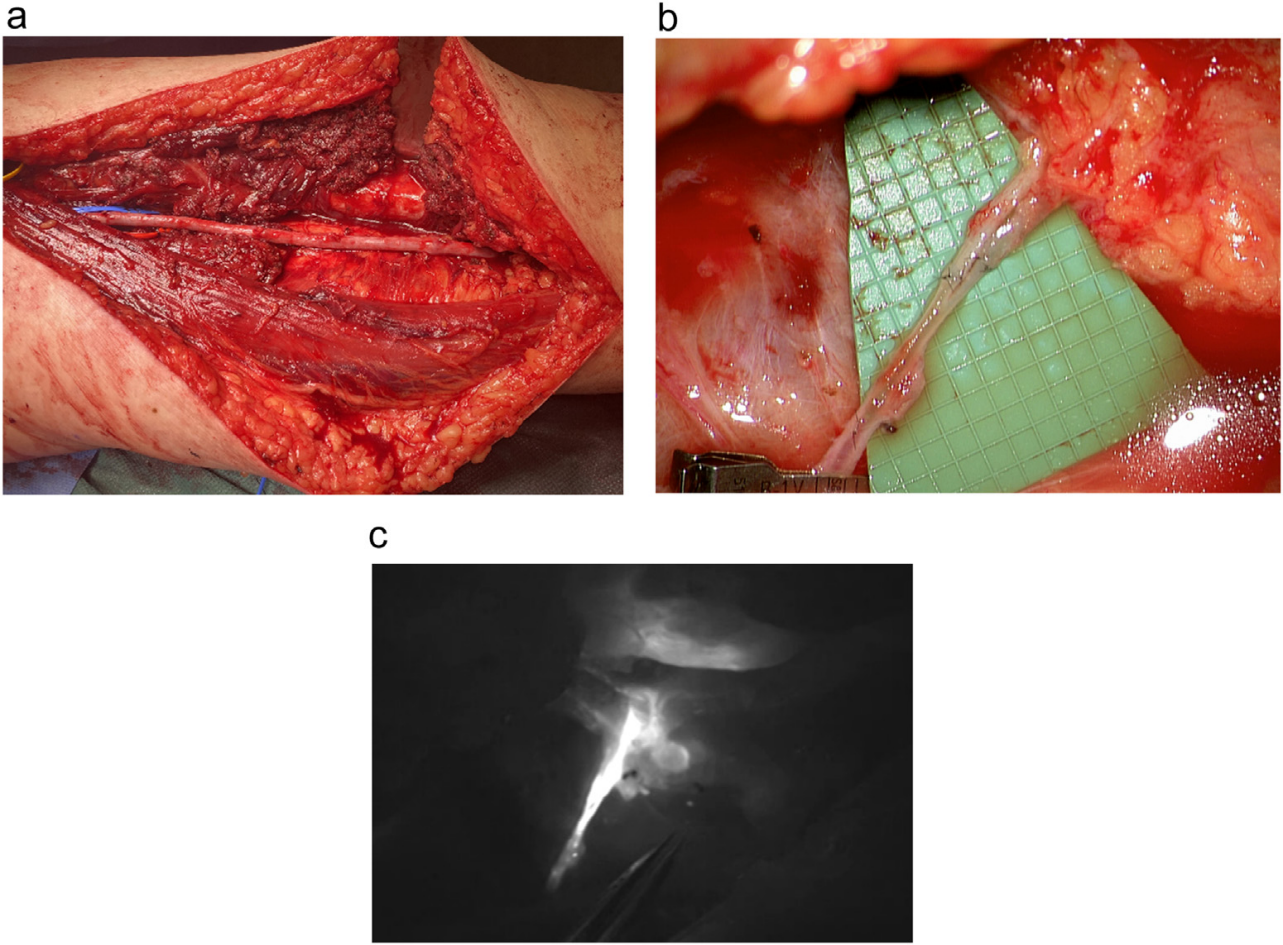
(a) Large wound cavity after sarcoma resection of the proximal medial thigh and reconstruction of the superficial femoral vein with a saphenous vein graft. (b) Robot-assisted LVAs in the depth of the wound. (C) ICG angiography showing good patency of the anastomoses.

The robotic systems have proven to be particularly suitable for performing LVAs of vessels with significant size mismatch (Figure 6) or preventive LVAs deep in the proximal thigh after sarcoma resection (Figure 7). The most common complication was seroma formation in four patients mostly after sarcoma resection, which was not linked to the use of the Symani Surgical System®, but the surgical procedure itself.

## Discussion

We present the first 21 patients in whom microsurgical procedures have been performed with the Symani Surgical System® in our unit. The feasibility and safety of using this device for microsurgery in humans have been previously demonstrated by our group. A further step is to assess the potential and the challenges of this innovative tool^2^. It is well known that microsurgery requires a specific set of skills, but despite the extensive training, there are factors intrinsic to the human hand, like tremor, precision, and accuracy, that are subjective and can be influenced by the psychological and physical status of the surgeon^6^. Robots have the capability of overcoming these limits. Furthermore, their performance is reproducible without the influence of external factors. The machine, for instance, does not experience physical tiredness, nor twitching and is able to hold a certain position indefinitely. These characteristics make it ideal for both reconstructive surgery and for operations where supermicrosurgical skills are required, such as in lymphatic surgery, perforator-to-perforator anastomoses, or distal replantations.

The Symani Surgical System® is solely designed for microsurgery and able to operate without fixed joysticks, which unties the surgeon from any contact with the operating table, thus leaving more space for other operators to be in the field. This characteristic combined with the capability of being teleoperated allows a second team to potentially operate on the patient. Furthermore, consisting of flexible robotic arms that are not connected to a fixed scaffold, it can reach into deep areas and to anatomic regions that would be difficultly accessible for the human hand, such as deep structures in the groin, axilla, or the thoracic and abdominal cavities. We performed LVAs of the thoracic duct in patients with chylous leaks or protein-losing enteropathy due to congenital malformations of the central lymphatic system, which usually requires to perform microsurgery deep within the thoracic or abdominal cavities. Based on our surgical experience, the Symani Surgical System® would have been of noticeable help in performing these complex surgeries in a not easily accessible location^3^. Additional applications of the robot for such surgeries will be further explored by our group.

To evaluate the accessibility of the robot and implementation into daily surgical practice, we evaluated the potentially biggest limitation - the learning curve. One of the most important factors to be considered is the robot`s intuitive use and the steepness of the learning curve. In order to explore, this further we divided the patients in two consecutive groups. There was a steady decrease in the time needed to perform the anastomosis that was statistically significant. In the first operations, the anastomosis took noticeably longer time when compared to the standard hand-sewn technique. In the last operation, two anastomoses were performed with the Symani® and one with the hand-sewn technique, and the times were almost comparable. The advance was not limited to the objective improvement. There was also a subjective perception technique improvement after several surgeries. Moreover, the ability to operate with the robot did also not diminish after several weeks of not operating due to absences from the clinic, indicating the ability of the brain to recover the newly learned skills.

Drawbacks that need to be considered when implementing the use of such external tools are time loss in preparing the surgical field, with the need for skilled personnel that is trained to adequately set it up in a timely fashion, as well as the time needed for its draping and the need to suspend the surgery to swing in and out the robot. These factors increase operational costs and surgery length, thus also partly prolonging the overall amount of anesthesia time for the patient. Other points that should be considered are the need for a bigger surgical room that can hold the robot and that allows the team to freely move around it. Because of the aforementioned considerations, the time necessary and the amount of space it requires, the surgeon should have the robot set up and ready before preparing the surgical field, making it difficult to decide for an improvised use when the surgery has already started. Other difficulties that have been noted while using it include technical problems with the instruments and their becoming sticky from the blood staining, which made them adhere to the sutures. This sometimes led to tearing of the sutures or to troublesome cutting. These difficulties can be overcome by frequent instrument rinsing and by having a scrubbed-in assistant who can aid in holding the tissues or the suture ends, but these solutions do add time loss to overall operation. In addition, during the course of the learning curve and the use of different badges of instruments, stickiness was not relevant issue anymore. Since the robotic system is working with a magnetic field to translate the surgeon`s movement into the operative field, interference with the operating table can occur. Therefore, it is advisable to place the patient at the side of the table to use operating tables with as little metal as possible and to switch the electronic column off when using the robot.

A limit of this study is that the robot was used by a very experienced surgeon, who already mastered the microsurgery technique and thus the learning curve would probably not be as steep for less experienced operators. In a next step, we plan to determine the learning curve for surgeons of different skill levels.

Based on our current experience, it can be expected that the Symani Surgical System® will not only aid already skilled surgeons in performing more complex surgeries (e.g., perforator-to-perforator anastomoses, distal replantations, operations in body cavities, etc.), but that it will also improve the accessibility to microsurgery for younger microsurgeons. Larger patient cohorts with longer investigation periods and inclusion of surgeons at different training levels will be necessary to investigate whether robotics in microsurgery will be advantageous in everyday procedures, e.g., free flaps or should be reserved for selected cases.

## Funding

No funding has been provided to perform this study.

## Ethical approval

The study is conformed to the World Medical Association Declaration of Helsinki and subsequent amendments and was approved by the Cantonal Ethics Committee of Zurich (BASEC approval number 2021-02351).

## Conflict of interest statement

Nicole Lindenblatt acts as a symposium speaker and clinical advisor for Medical Microinstruments.

All other authors have no conflict of interest.

## Appendix Supplementary materials

Image, video 1
